# Long-Term Effects of Balance Training on Habitual Physical Activity in Older Adults with Parkinson's Disease

**DOI:** 10.1155/2019/8769141

**Published:** 2019-08-07

**Authors:** Håkan Nero, Erika Franzén, Agneta Ståhle, Martin Benka Wallén, Maria Hagströmer

**Affiliations:** ^1^Department of Neurobiology, Care Sciences and Society, Division of Physiotherapy, Karolinska Institutet, Alfred Nobels Allé 23, 14183 Huddinge, Sweden; ^2^Function Area Occupational Therapy & Physiotherapy, Allied Health Professionals Function, Karolinska University Hospital, Stockholm, Sweden

## Abstract

The HiBalance program is a progressive and highly challenging balance training intervention incorporating Parkinson's disease (PD) specific balance components. The program improves balance and gait and increases the amount of ambulation in short-term, in older adults with PD. Yet, potential short- and long-term effects on habitual physical activity and sedentary behavior are currently unidentified. The aim of this study was to conduct preplanned secondary analyses of short- and long-term effects of the HiBalance program on objectively measured amount and bouts of brisk walking, sedentary behavior, and total physical activity in older adults with PD. Further, our aim was to investigate demographic, intervention-related, disease-related, and function-related factors potentially related to a difference in activity after intervention. A total of 100 older adults with mild-moderate PD were recruited. The intervention group participated in the HiBalance program, and the control received care as usual and was offered the HiBalance program after study termination. Physical activity data were collected using accelerometers at baseline, after intervention and after 6 and 12 months. A multilevel model was utilized to investigate the postintervention and long-term (6 and 12 months) effects on total physical activity, amount and bouts of brisk walking (i.e., moderate intensity physical activity), and sedentary behavior. Between-group difference for the main outcome brisk walking was at postintervention: Δ −10, CI −23.78 to 3.69 min/day (*p* < 0.05); 6 months: Δ −10, CI −23.89 to 3.89 min/day (*p* < 0.05); and 12 months: Δ −4, CI −16.81 to 8.81 min/day (*p*=0.43). Being part of the intervention group as well as finishing training during spring/summer showed an independent association to increased brisk walking after the intervention period. In conclusion, the HiBalance program increases the physical activity on moderate intensity after intervention and at 6 months but not at 12 months, independently of improved balance. Season seems to influence the effect on the physical activity.

## 1. Introduction

Parkinson's disease (PD) is a neurodegenerative disease with symptoms such as tremor, rigidity, bradykinesia, and impaired postural stability (balance). Due to the progressive nature of PD, balance impairments gradually increase in severity, which leads to falling [1], fear of falling [2], and a recession in physical activity (PA) [3, 4]. For older adults in general, reaching the recommended level of PA may increase the chance of healthy ageing, and if the recommendations cannot be reached, any reduction in sedentary behavior or increase in PA has beneficial effects on health, quality of life, risk of noncommunicable disease, and functional limitations [5]. Despite the difficulty of defining a minimum intensity threshold at which PA may be considered health-enhancing, it seems that PA of at least moderate relative intensity is effective. As such, walking is one form of activity that leads to decreased risk of all-cause mortality, and for older adults, research suggests a threshold of at least moderate activity to maintain functional independence [6]. Hence, to increase the life span and improve the health in older adults with PD, it is essential to reverse or decelerate the negative trend of a sedentary lifestyle and increase PA [7–9].

Falling is common in PD, and it has been estimated that about 18 to 65 percent of afflicted are frequent fallers [10]. Frequent fallers tend to have a greater deal of activity limitations in daily living, thereby a lower ability to perform common day-to-day activities and also a greater fear of falling, which has been shown to be associated with lower levels of habitual PA [11]. Hence, if balance impairment and fear of falling persist or even escalate, it may hinder older adults with PD to increase their PA, even if motivation to do so exists. Fortunately, there is evidence of beneficial effects of balance training on gait-related activities [12–14].

The HiBalance program is a progressive and highly challenging balance training intervention incorporating PD-specific balance components [15]. Results from a randomized controlled trial performed by our research group has shown that compared to a control group, not only balance the performance, step length, walking speed, and ability to perform activities in daily living but also number of steps per day increased in older adults with PD after completing the 10-week intervention [13], and the long-term follow-up showed that the training effect on balance performance diminished within six months [16]. Nonetheless, the short- and long-term effects of the HiBalance program on more detailed measures of PA and sedentary behavior are still not fully investigated. As an example, it is still unknown whether the increase in activity was performed in bouts or was spurious, which is of interest since recommendations state that health-enhancing PA should be performed in a minimum of 10-minute-long bouts [17, 18]. Neither do the results reveal the intensity of the ambulatory activity or its duration [19]. Adding on, as suggested by the preliminary results, long-term analysis may help in deriving the necessary intervals of the balance training program, in order to keep the potential beneficial effect on PA and sedentary behavior. Hence, a greater understanding of possible health benefits, as well as clinically valuable knowledge, may be uncovered by further exploration of the data.

Therefore, the aim of the present study was to conduct the secondary analyses investigating postintervention and long-term (6 and 12 months) effects of the HiBalance program on objectively measured amount and bouts of brisk walking, amount and bouts of sedentary time, and total PA. We hypothesized that the improved balance and walking speed previously found [13] also increased objectively measured habitual PA in general and time spent on health-enhancing levels specifically, in the postintervention and long term, respectively.

It is also of interest to investigate whether the increase in PA after the intervention is linked to improved balance, since it has been suggested that improved balance performance is associated with a greater ability of being more active [11, 20, 21]. Further, if a balance intervention increases health-enhancing PA, it may also be advantageous to assess whether there are other potential demographic, study-related, disease-related, and functional factors that modify this effect. Hence, as a secondary aim, this study also wanted to investigate factors potentially related to a difference in activity after intervention.

## 2. Materials and Methods

### 2.1. Study Design

This study reports preplanned secondary analyses on habitual PA and sedentary behavior of a randomized controlled trial (RCT) of the BETA PD-project aimed at investigating the effects of the HiBalance program (clinical trial number NCT01417598).

### 2.2. Participants

A total of 100 individuals with PD and impaired balance (based on a clinical assessment), were included [13]. All included participants had a clinical diagnosis of idiopathic PD (Hoehn & Yahr scores 2-3), were >60 years old, and had no other existing neuromuscular disorders, including severely flexed posture. In addition, the participants had no history suggesting atypical PD symptoms and had been clinically diagnosed according to the Queens Square Brain Bank criteria [22], whilst having the ability to ambulate indoors without the need for a walking aid. Further, eligible participants had ≥3 weeks of stable dopaminergic medication.

Recruitment comprised three waves (three sets of both intervention and control groups), where two waves started the intervention during spring and one during fall. Both groups were assessed using all the included measures at baseline and after intervention, as well as 6 and 12 months thereafter.

The participants were randomly assigned in blocks of four to either the intervention group participating in the HiBalance program, or the control group [13, 15]. Researchers were blinded to group allocation at baseline assessments but not at follow-up assessments. During follow-up, participants were never assessed by a researcher that had been involved in the training. Ethical approval was obtained from the Regional Board of Ethics in Stockholm, Sweden (Dnr 2006/151-31, 2009/819-32, and 2011/37-32), and all participants provided written informed consent before inclusion.

### 2.3. Intervention

The HiBalance program contained a 10-week, three times/week, balance training intervention [13, 15]. Each session lasted 60 minutes, and the group (4 to 7 participants per group) was led by two trained and experienced physiotherapists. The intervention followed a predefined framework based on motor-learning principles and was of a progressive nature where difficulty increased each week [15]. Participants in the control group were encouraged to maintain their normal physical activities and not restricted from participation in ongoing rehabilitation programs and were offered the HiBalance program after study termination. All participants were advised to keep up their normal level of exercise throughout the intervention period.

According to the clinical procedure, all participants allocated to the intervention group were given PA on prescription (PAP) at time of the postintervention measurements. The recommended activity was based on current PA recommendations for health (WHO) and their interests, needs, and abilities. In addition, participants were supported to set specific, measurable, attainable, realistic, and time-given goals for physical activity and given a brochure with information on the benefits of physical activity. The PAP was followed up telephonically after three months, by a physiotherapist asking the participant questions regarding compliance to the PAP, performed activity type, frequency, and duration.

### 2.4. Assessments and Outcomes

All assessments were conducted by experienced physiotherapists after a predesigned protocol. Participants were informed about the study, and informed written consent was obtained. The subsequent test procedure included clinical tests of gait and balance, followed by an interview of questionnaires and distribution of accelerometers. Half of the participants did the physical tests first and the other half the interview first. Finally, instructions were given on how to wear the accelerometers during the following week for measuring habitual PA.

Habitual physical activity and sedentary behavior data were collected using the accelerometer Actigraph GT3X+ (ActiGraph, Pensacola, FL). GT3X+ is an accelerometer that records changes in movement over time (acceleration) in three axes expressed as an arbitrary unit named counts. Participants were assigned the accelerometer attached to a belt and instructed to wear it around the hip and positioned lateral to the spine, for the duration of seven days, only removing it for showering, swimming, and bathing and at night. Participants received oral and written information on how to use the accelerometer and were also asked to fill in a wear-time diary during the period and told that the monitor measures daily movement. After the measurement period, the accelerometer was returned in a prepaid padded envelope.

The data were filtered, cleansed, and computed using ActiLife v.6.13.3 (ActiGraph, Pensacola, FL). A 15-second epoch was used, where ≥90 minutes of consecutive zeroes was defined as nonwear time and discarded. Spike tolerance and small window length were set to 2 minutes and 30 minutes, respectively [23]. Normal band-pass filter was utilized [24], and a minimum of 540 minutes/day and four days/week was used as thresholds for valid data [25–27]. As the primary outcome, minutes spent at or above 328 counts/15 sec in the vertical axis, corresponding to walking at a speed of >1.0 m/sec (3.6 km/h), was considered as minutes of brisk walking, based on a previous calibration study on an older adult PD population [28]. Brisk walking is considered to be equivalent to at least moderate intensity [5, 28]. Sedentary time was based on accelerometer cut-points derived from older adults in free-living determined by Aguilar-Farías et al. [29]. To calculate bouts of brisk walking and sedentary time, a 10-minute bout threshold was used [30]. Amount of sedentary time per day was adjusted for wear-time per day and presented as percentage of wear time. Total activity counts (TAC) of the vector magnitude was used as a representation of total PA/day [31]. Missing accelerometer data were defined as data lost due to follow-up, while all data not reaching the cutoffs of defined wear time were considered invalid.

#### 2.4.1. Covariates

Factors that can influence the effect of the program based on the previous studies and clinical reasoning such as demographic factors, disease-related factors, and motor function, collected at baseline, were included. Demographic data such as age, sex, and BMI of the participants were collected using structured questions.

Proxys for disease-related factors were fall history, the motor performance, and activity of daily living (ADL) part of the Unified Parkinson's Disease Rating Scale (UPDRS) [32, 33], and levodopa equivalent dose (LED) [34].

Proxys for motor function was balance control, gait velocity, and step length. Balance control was assessed using the Mini-BESTest, [35]. Improved balance after intervention was defined as an increase of ≥3 points on the Mini-BESTest [36]. Assessment of normal gait velocity (self-selected) and step length was performed using a GAITRite electronic walkway system (CIR Systems, Inc., Havertown, PA, USA) [13, 15].

Due to the fact that the season during training varied between waves of participants in the HiBalance program (participants finished training during May-June or in December) and since the Swedish climate entails quite cold winters and there is evidence of a seasonal effect of poor or extreme weather on amount of PA [37], it was of interest to investigate whether season during intervention had an independent effect on PA. Calendar month defined the season at postintervention (spring/summer or fall/winter).

### 2.5. Sample Size

The main focus of the RCT was postintervention with a secondary aim to study the long-term effect. Hence, the power calculations were primarily conducted for postintervention. Sample size, detailed in the study protocol, was based on the previous calculations based upon a feasibility study on the outcome measures of balance control, gait velocity measured in a movement laboratory, and concerns about falling and steps per day [15]. In order to achieve 80% power with a 2-sided *α* level of 5%, the number of subjects required per group and the hypothesized effect size, respectively, were 24 (effect size = 0.83) for Mini-BESTest, 27 (effect size = 0.83) for gait velocity, 32 (effect size = 0.71) for FES-I, and 19 (effect size = 0.79) for steps per day. Taking an anticipated dropout rate of 15% into account, a sample rate of 40 in each group was needed. Because of long-term follow-up and risk of further dropout, the group size was increased to 50 subjects per group.

### 2.6. Data Analysis

Demographic data together with disease-related factors and function-related factors were summarized for descriptive purposes. Missing data analysis of outcome variables was performed using Little's test, a single global test for investigating whether missing values of multivariate data are missing completely at random or depends on the variables in the data set [38].

Between-group differences were calculated and presented as mean and confidence interval (CI). To investigate the postintervention and long-term effects of the intervention on PA (TAC, minutes of brisk walking and bouts of brisk walking) and sedentary behavior, a multilevel model (mixed effect model) was utilized. This model does not rely on listwise deletion; therefore, all available information even if values are missing is used. Time, group affiliation, time *∗* group, and season (control factor) were set as fixed factors, and the intercept of each individual as well as time were set as random factors, thereby allowing variation in levels of PA at baseline and the effect of time. Repeated covariance type was set to a heterogeneous first-order autoregressive structure, taking into account the higher correlation between measures closer in time compared to those further apart [39]. Effect sizes for each time point were calculated using Cohen's effect size (Cohen's *d*), based on between-group differences.

Further, a multiple linear regression was performed to investigate the factors associated with the (absolute positive or negative) difference in participants' amount of brisk walking (subtracting baseline values from postintervention). Group affiliation, season, and improved balance were entered (all dichotomous), and age was controlled for. In the second step, the potential interaction between group affiliation and season (group *∗* season) was also added. The statistical analyses were performed in SPSS v.23 for Windows (IBM SPSS Inc., Chicago, IL, USA) and R (R Core Team (2015). https://www.R-project.org/).

## 3. Results

**Figure 1:**
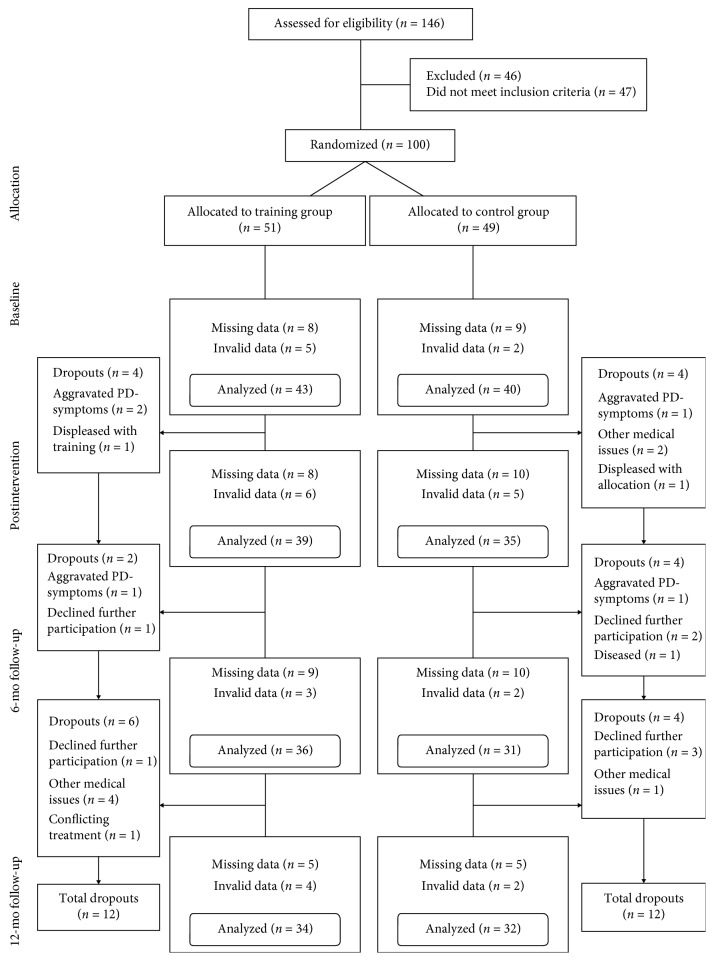
Flow chart of physical activity data from baseline to the 12 months follow-up.

Figure 1 describes the participant flow in relation to the outcome physical activity. The amount of valid accelerometer data from participants varied between measurements, from 43 and 40 in the intervention and control groups at baseline to 34 and 32 at the 12-month follow-up. The percentage of total missing physical activity data (independent of group affiliation and including dropouts) at baseline, after intervention, and long-term (6 and 12 months) were 17%, 26%, 33%, and 34%, respectively. At the final time point (12 months), the total number of dropouts was 24. According to Little's test, all missing data were missing completely at random (*p* < 0.05 for all).

After exclusion of participants not reaching the set cutoffs for valid data, a total of 83 participants with data from baseline and 74 from postintervention remained (16 and 20 women in the intervention and control groups, respectively). Participant characteristics are presented in Table 1.

### 3.1. Postintervention and Long-Term Effects

Table 2 presents the outcome variables for all four measurement periods for the control and intervention groups. Between-group difference for the main outcome brisk walking at postintervention was Δ −10, CI −23.78 to 3.69 min/day; for 6 months: Δ −10, CI −23.89 to 3.89 min/day; and for 12 months: Δ −4, CI −16.81 to 8.81 min/day.

The postintervention and long-term (6 and 12 months) effect analysis using a multilevel model resulted in an overall significant effect of time and an interaction effect between group and time (*p* < 0.05) for minutes of brisk walking, in favour of the intervention group. Furthermore, there were significant interaction effects of group and time when comparing baseline to postintervention as well as baseline to the 6-month follow-up (*p* < 0.05). The effect dissipated when comparing baseline to the 12 months follow-up (*p*=0.43). This pattern was also visible when inspecting the trajectory of both groups visually (Figure, supplementary material (available here)). Effect sizes (Cohen's *d*) for the between-group comparisons of brisk walking at postintervention, 6 months, and 12 months were 0.34, 0.36, and 0.15, respectively. No other PA variables showed a significant interaction effect of group and time, although total PA and sedentary time showed a significant overall effect of time (*p* < 0.01), i.e., reduced PA and increased sedentary time.

### 3.2. Factors Associated with an Activity Difference

The multiple linear regression investigating associated factors to a difference in minutes of brisk walking from baseline to postintervention resulted in a significant model (adjusted *R* ^2^ of 0.24). The model proposed that performing the training during spring/summer and being part of the intervention group were both independently associated with an increase in minutes of brisk walking (Table 3).

## 4. Discussion

The short- and long-term effects of the HiBalance program have been investigated previously, and in the present study, we performed an in-depth analysis of the effects of the intervention on habitual PA, on postintervention as well as long-term basis. The combined evaluation displayed positive interaction effects on minutes of brisk walking, and one explanation can be that this finding signifies an intermittent increase in activity in daily life, which could carry over to an improved ability of, or interest in, taking part in societal and social activities, potentially increasing the physical function and improving the quality of life. But, to validate these assumptions, further research is needed.

The increase in brisk walking by 10 minutes per day at postintervention and at 6 months corresponds to 70 minutes increase per week, which is half of the recommended weekly dose of physical activity [17, 40]. This dose has shown the potential to give health benefits in adults and most likely also beneficial in the elderly with PD [41]. The increase in minutes of brisk walking in free living but lack of effect on effect on free-living sedentary behaviour, total PA, and bouts of brisk walking can have several explanations. For example, it may be due to a high variability within the groups, since absolute mean values of sedentary and total PA showed a difference between groups, in favour of the intervention. This might be due to the fact that PD is a heterogeneous disease with diverse symptoms and a course of progression that varies across individuals.

The long-term perspective proposed that the effects on PA dissipated somewhere between six and 12 months after the intervention, and the intervention group returned to levels in line with those at baseline and the control group. Furthermore, spring/summer season as well as intervention group affiliation were associated with an increased amount of minutes of brisk walking after intervention. However, improved balance control remained statistically insignificant, suggesting that it is not an improvement of balance per se that might explain the increase of PA after the training period, but rather being part of an exercise intervention.

The results showing that the long-term effect on brisk walking dissipated after six months were rather expected. Previous investigations have presented results showing a recession in PA long term, after an intervention [42, 43]. However, there is also evidence of the opposite, showing increases in the proportion of participants meeting the recommended amounts of PA after intervention. Yet, it has been suggested that some form of intervention booster or tailored exercise prescription needs to be added to improve the uptake [43]. The current study's intervention included a PAP, but since the study design did not incorporate a third group performing training without receiving the prescription, it is unknown whether the PAP had any supplementary effect to the balance training, or whether the effect on PA might have dissipated earlier without PAP. According to interviews of study participants, the goals set in the PAP had rarely been achieved [44]. Although it was frequently expressed that setting physical activity goals upon program completion was important, these goals appeared, in general, not to have been achieved. For this reason, many participants strongly expressed the need for repeated group programs to account for this disease-related apathy [44]. Reduced initiative was also more evident when the group-based context ceased.

Since walking outdoors is the most common type of activity undertaken amongst older adults [45], weather, temperature, and day length may have an influence on their level of daily ambulation [46]. As such, winter and a cooler temperature may deter older adults from activity. Sweden is located in the northern hemisphere with a climate composed of cold winters, and icy roads may increase the risk of falling, especially for a population already suffering from a disease linked with an increased fall-risk. According to our clinical experience, it is not uncommon for people with PD to be afraid of walking outdoors during winter, due to this fact. Thus, the association between an increase in brisk walking and exercising in spring/summer reported here is not surprising. In the current study, season had an independent effect on the level of brisk walking separate from the effect of the intervention. Yet, it is suggested that this association is further investigated before specific recommendations to clinicians are made based on this specific finding.

The results further suggested that there was no independent association between change in PA and improved balance control, even though the multilevel model implicated that the intervention led to a higher amount of minutes of brisk walking. Further investigation into what other factors or mediators might have led to the increase in brisk walking was not included in the scope of this study, although the level of explained variance suggests there could be several more. However, not only PD and its related symptoms but also the ageing process, and with it, the associated reduction in higher intensity activity and muscle mass, may contribute to a decline in aerobic capacity [47, 48]. Considering the results presented above, the 10 week, three times a week, one hour per session- progressive program presumably promoted the participants' physical fitness, which in turn may have led to the desirable consequence of increased ambulation in daily living. Furthermore, results regarding the relationship between balance performance and PA found in the literature, have been contrasting. Although a link between the sedentary status of older adults and poorer postural balance has been reported [49, 50], there is research performed on frail elderly reporting no correlations between postural balance and ambulatory performance [51]. Further, it has been suggested that exercise may reverse recession in balance performance [52], proposing an inverse causational direction. Or, perhaps the divergence in results is due to differently measured aspects of PA between the current study and previous research. Whatever the cause, the relationship is in need of further evaluation.

The study encompassed some limitations. Power was primarily calculated for detecting a difference before and after the intervention with regards to balance and gait measures separately not for multiple outcomes. In addition, the power for PA was calculated based upon a pilot study, and in the actual RCT, the variance of PA data was larger than expected. The study is therefore most likely under-powered. Since participants were recruited based on a clinically assessed need for balance training and being of mild-to-moderate disease severity without cognitive decline, the ability to generalize the results is restricted to this subgroup. Lack of blinding and a nonactive control group are limitations as some of the observed effects could be attributed to a nonspecific effect as opposed to the training. This should be considered when interpreting the results. Furthermore, the amount of missing data due to dropouts and invalid activity data may have influenced the result. Such an example is the analysis of the measure of total activity, showing an increase in the intervention group compared to the control, yet not reaching significance. Future studies should employ greater sample sizes and/or avoid missing data, if further investigation into effects on measures of PA levels is of interest. Adding on, according to the analysis of activity difference between baseline and postintervention, an increase in brisk walking was not independently related to improved balance control. Hypothetically, on top of the heterogeneous population, there could be other factors (e.g., behavioural and mental) related to an increase in brisk walking that is not measured herein. In addition, disease-related factors included might not be sensitive enough and behavioral and mental factors such as exercise self-efficacy, motivation, and previous experiences with exercise were not collected in this study.

As mentioned above, the added effect of PAP is unknown and needs to be further investigated in the future trials. However, the feedback from participants on the PAP emphasizes that tools that support management of physical activity in relation to the disease are needed. Participants might not be ready to change or have the tools for self-management and might therefore need further and deeper support in this matter.

## 5. Conclusions

The results herein indicate that being part of a balance intervention increases physical activity on moderate intensity (brisk walking), but has no effects on sedentary behavior and total PA. Furthermore, the long-term analysis showed a dissipation of the effect on time in moderate intensity before 12 months, with a return to levels similar with those at baseline. This, together with results showing that season had an independent effect of the intervention, may help clinicians plan for extra support such as a booster session if the balance training period ends in the winter and repeated or continued.

## Figures and Tables

**Table 1:** Demographics of subjects with valid baseline accelerometer data separated by groups.

Characteristics	Intervention (*n* = 43)	Control (*n* = 40)	*p*
	Mean (SD)	Mean (SD)	
Age (years)	72 (6)	74 (6)	0.25
Male/female (*n*)	27/16	20/20	0.24^a^
BMI (kg/m^2^)	25 (4)	25 (5)	0.91
LED^1^	584 (290)	649 (420)	0.93^b^
UPDRS^2^ motor	37 (11)	37 (11)	0.86
UPDRS^2^ ADL	15 (4)	13 (5)	0.16
Mini-BESTest	19 (3)	18 (3)	0.15
Gait velocity (m/s)	1.19 (0.20)	1.15 (0.18)	0.39
Step length (cm)	63 (10)	61 (8)	0.16
PD^3^ duration (years)	6 (5)	6 (5)	0.97^b^

Comparison between groups performed with independent samples *t*-test, unless marked otherwise. ^1^Levodopa equivalency dose. ^2^Unified Parkinson's Disease Rating Scale. ^3^Parkinson's disease. ^a^Pearson chi-square. ^b^Mann–Whitney *U*-test.

**Table 2:** Mean values of outcome variables for all four measurement periods for control and intervention groups, respectively.

	Baseline		Postintervention		6-month follow-up		12-month follow-up	
	Mean (SD)		Mean (SD)		Mean (SD)		Mean (SD)	
Variables	Intervention *n* = 43	Control *n* = 40	Intervention *n* = 39	Control *n* = 35	Intervention *n* = 36	Control *n* = 31	Intervention *n* = 34	Control *n* = 32
TAC^1^	280835 (134637)	292475 (154621)	316068 (175388)	289886 (147570)	288230 (126578)	268606 (133582)	270882 (147830)	253270 (126725)
Brisk walking^2^	32 (26)	32 (28)	37 (33)	27 (25)	36 (31)	26 (25)	30 (30)	26 (21)
Bouts of brisk walking^3^	0.79 (0.92)	0.74 (1.08)	0.81 (1.06)	0.55 (0.80)	0.98 (1.06)	0.60 (0.93)	0.77 (1.09)	0.51 (0.80)
Sedentary^4^	77% (9%)	74% (9%)	74% (10%)	74% (7%)	76% (8%)	76% (8)	78% (9%)	75% (9%)
Sedentary bouts^5^	14 (3)	12 (3)	13 (4)	12 (3)	14 (3)	14 (3)	15 (4)	13 (3)

^1^Total activity counts per day. ^2^Minutes of activity corresponding to brisk walking per day (>1.0 m/s). ^3^Number of bouts of ≥10 minutes of activity corresponding to brisk walking per day. ^4^Percentage sedentary of total wear time per day. ^5^Number of bouts of ≥10 minutes of sedentary per day.

**Table 3:** Multiple linear regression model-associated factors to difference in amount of brisk walking (min/day) between pre- and postintervention (*n* = 66).

Correlates	*B*	Beta	*p*	95% CI^a^ for *B*	
				Lower	Upper
Constant	−5.32		0.86	−64.62	53.99
Group^b^	−20.15	−0.52	<0.01	−31.82	−8.47
Age	0.30	0.08	0.47	−0.52	1.11
Season^c^	−19.05	−0.48	<0.01	−31.21	−6.88
Group *∗* season	9.83	0.20	0.25	−7.23	26.88
Improved balance^d^	−5.13	−0.13	0.29	−14.79	4.54

^a^Confidence interval. ^b^Coded as 0 for intervention, 1 for control. ^c^Spring/summer = 0, fall/winter = 1. ^d^≥3 points of the Mini-BESTest total score.

## Data Availability

The data used to support the findings of this study are available from the corresponding author upon request.

## Supplementary Materials

Supplementary Materials Trajectory over time of minutes per day of brisk walking in free living, divided by groups (mean values (±95% confidence interval)).
