# Supplementary figures and images for: Long-Term Effects of Balance Training on Habitual Physical Activity in Older Adults with Parkinson's Disease
Source: Parkinsons Dis. 2019 Aug 7;2019:8769141. doi: 10.1155/2019/8769141 (PMC6702829; doi:10.1155/2019/8769141)

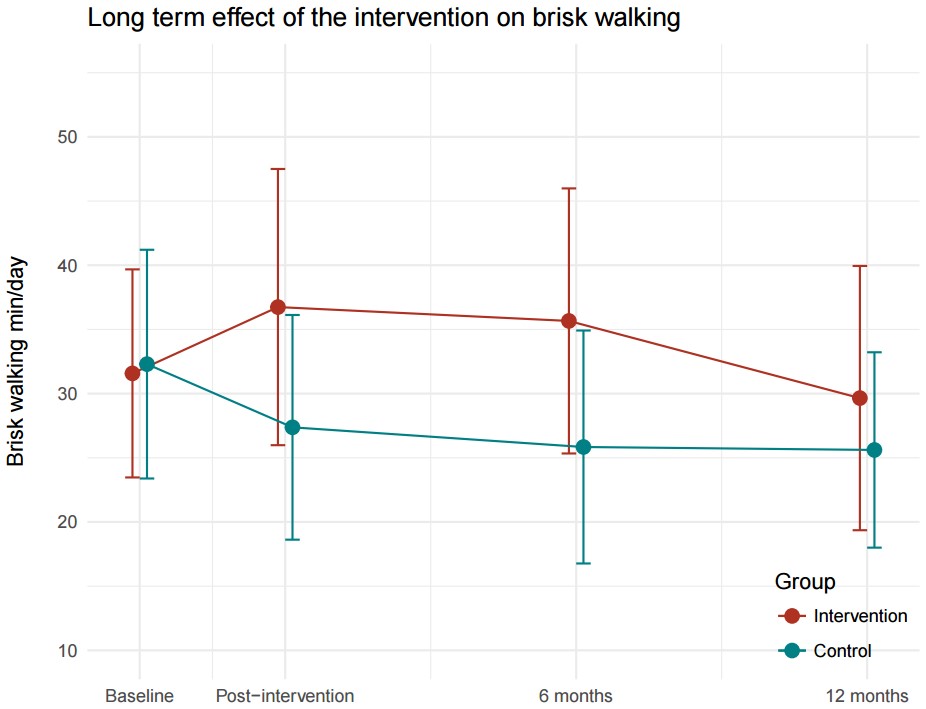

Supplement: Supplementary Materials — Trajectory over time of minutes per day of brisk walking in free living, divided by groups (mean values (±95% confidence interval)). [file 8769141.f1.jpg]
